# The Oceanus Borealis of ancient Mars

**DOI:** 10.1093/nsr/nwad146

**Published:** 2023-05-18

**Authors:** Victor R Baker

**Affiliations:** Departments of Hydrology and Atmospheric Sciences, Planetary Sciences, and Geosciences, University of Arizona, USA

Fifty years ago, the Mariner 9 spacecraft revealed that the Martian surface has a great planetary dichotomy separating heavily cratered southern highlands from a huge area of more lightly cratered northern plains. Following this discovery, informal hypothesizing by geologists considered the possibility of an Earth-like water body occupying the northern lowland Martian plains. As reviewed by Xiao *et al.* [1], this initial thinking led to a succession of published papers in the 1980s. Considerable evidence was also accumulated for ancient water-related phenomena on the planet [2] and, in 1991, the related implications for an Earth-like global hydrological cycle was recognized as being closely tied to the ancient northern plains water body, named Oceanus Borealis [3].

For the past three decades the Oceanus Borealis concept has engendered scientific controversy as an ‘outrageous geological hypothesis’. The classical sense of that designation, as originally formulated by William Morris Davis [4], characterized such hypotheses as inferences that should not be accorded ‘...a brief contemplation followed by an off-hand verdict of “impossible” or “absurd”’. Instead, their contemplation should be ‘...deliberate enough to seek out just what conditions would make the outrage seem permissible and reasonable’.

To claim, as some philosophers of science do, that hypotheses are merely propositions to be verified or falsified is to advocate for an end to further scientific inquiry into the relevant matter. A conclusion of truth or falsehood produces the certainty that nothing new will be discovered. Geological hypotheses, by contrast, are ‘working’ [5]; they function to direct an on-going pursuit of new evidence. They are also fallible, but they focus on potentially productive investigations [6] into what nature presents.

Results presented by Xiao *et al.* [1] are exemplary of this geological reasoning process. The Tianwen-1/Zhurong rover encountered boulder-sized samples of the Vastitas Borealis Formation (VBF) that signifies the depositional processes that occurred in Oceanus Borealis. The sedimentological interpretations made from these boulders rely on analogical reasoning [6], of a kind central to geological investigations [7], comparing the observed sedimentary character of the Martian examples to that associated with well-understood depositional environments on Earth. Specifically, a case is made for deposition in a marine environment, with the best comparison seeming to be with shallow marine counterparts on Earth.

While geological reasoning involves productive, working hypotheses, these are always tentative. This leads to a need for multiple such hypotheses, and these can also work together to produce a regenerative view of the original concept [5]. Two such alternative working hypotheses might involve other forms of marine sedimentation. One possibility could be the deep-water facies of the mega-tsunami phenomena that Xiao *et al.* [1] note have recently been recognized from their runup features along the margins of the VBF, locally complicating interpretations of the shorelines of Oceanus Borealis.

Another working hypothesis might involve the high-energy mega-flooding that produced the immense outflow channels that were discovered early in the modern era of Mars exploration [8] and were subsequently recognized as major factors for initiating the later phase of Oceanus Borealis formation [3]. On this view the boulder sedimentology might result from turbidite-like density flows that moved across the floor of Oceanus Borealis from entering sediment-laden hyperpycnal flows that would have been generated by the Martian mega-flooding [9]. Past high-energy mega-flooding on Earth emplaced such deposits over distances of thousands of kilometers across the Pacific Ocean floor [10].

One reason the Oceanus Borealis hypothesis seemed so outrageous to planetary scientists is that physical climate modeling has encountered considerable difficulty in predicting the appropriate conditions that could transform ancient Mars to a prolonged period of Oceanus Borealis global hydrological cycling from what today is an extremely cold, dry land surface with such low atmospheric pressure than any liquid water would rapidly freeze or sublimate. However, these models involve human assumptions about how Mars works (combined with known physical laws) to extrapolate backwards from the current state of Mars to a hypothetical ancient state. Mars evolves through real time in an opposite direction, through a continuity of temporal change from conditions in its deep past to the conditions of today. We must learn about such evolution by investigating the evidence that Mars presents to us, as exemplified by the discoveries reported by Xiao *et al.* [1]. Our understanding of Oceanus Borealis must continue to be informed by similar geological investigations.
